# Power shapes power construal: the mediating role of entitlement

**DOI:** 10.3389/fpsyg.2024.1459405

**Published:** 2024-12-18

**Authors:** Xinyue Wang, Jingyuan Liu, Hong Li

**Affiliations:** Department of Psychology and Cognitive Science, Tsinghua University, Beijing, China

**Keywords:** power, power construal, personalized, socialized, psychological entitlement

## Abstract

**Introduction:**

This article investigates the effect of power on power construal through psychological entitlement as a mediator across three empirical studies (*N* = 895).

**Methods:**

We examine how people in powerful and powerless conditions construe power, with psychological entitlement as a key mediator.

**Results:**

We uncover a nuanced association whereby people in powerful conditions predominantly adopt socialized orientations to construe power, whereas in powerless conditions, people tend to construe power as a paradox. These different construals appear to be significantly mediated by the perceived psychological entitlement of powerful people.

**Discussion:**

Our research provides insights into the nature of power by revealing two interesting phenomena: the powerful people prosocial gloss and the powerless people paradox. By extending the theory of culturally nurtured power concepts to include the impacts of power itself on power construal, our research offers insights into how power is construed differently based on one’s power position (i.e., with or without power), enriching our understanding of power. This exploration broadens theoretical frameworks and implicates practical considerations in organizational and social settings.

## 1 Introduction

Power construal, encompassing views on what is desirable and meaningful to do with power (Torelli and Shavitt, 2010), plays a pivotal role in effective management, government, and social systems (Barkow, 1989; Boehm, 1999; McClelland, 1973; Van Vugt et al., 2008; Winter, 1973). Despite its importance, fundamental questions remain regarding how power influences power construal. Specifically, do people construe power differently when they are in powerful and powerless conditions? Within the realm of social power (see Van Dijke and Poppe, 2006), researchers have extensively explored power conceptualization in terms of personalized (i.e., self-interested power, seeking self-centered goals, influencing others and garnering praise to enhance their status; Kipnis, 1976; Winter, 1973; McClelland, 1987; Maner and Mead, 2010; Miyamoto et al., 2018; opportunity, Sassenberg et al., 2012) versus socialized (i.e., prosocial power, pursuing prosocial goals, benefiting others and taking responsibilities to others; McClelland, 1973; Winter, 1973; Torelli and Shavitt, 2010; Miyamoto et al., 2018; responsibility, Sassenberg et al., 2012) orientations. Many previous studies have reported that powerful people often exhibit selfish actions aimed at personal goals and needs (Kipnis, 1976; McClelland, 1987; Winter, 1973, 1993), potentially resulting in antisocial effects (Cislak et al., 2018). However, few studies have investigated how powerful people construe power, and it remains an open question as to whether powerful people’s construals of power are likewise selfish or personalized. Associated with this, how powerless people construe power also remains an open question.

In this article, we extend the above issues to how power influences power construal. We examine power construal toward socialized versus personalized orientations and compare the construals of people in powerful and powerless conditions. Specifically, are powerful or powerless people more likely to construe power as socialized or personalized? This research addresses an important gap in the power literature. Previous studies have identified several factors that can shape people’s construals or concepts of power, among which culture is essential (Hofstede, 1980; Torelli and Shavitt, 2010). Cultural contexts not only shape concepts of power (Torelli and Shavitt, 2010), but also shape the perception and acceptance of power inequalities (Hofstede, 1980). Cultures with high power distance where power hierarchies are more rigidly accepted, may foster a more personalized power construal. In contrast, cultures with low power distance where equality is emphasized, may encourage a more socialized power construal (Hofstede, 1980; Torelli and Shavitt, 2010). Some other important power theories may also offer insights into a better understanding of power, for example, Leader-Member Exchange (LMX) theory, describing how the quality of relationships between leaders and subordinates, rooted in trust, reciprocity, and respect, influences organizational behavior and employee performance (Graen and Cashman, 1975; Graen and Uhl-Bien, 1995). However, these perspectives remain largely leader-centered and focus on the interaction quality between powerful and powerless, rather than directly address how power is construed by powerful and powerless people. The key factor has still been neglected: how does power itself influence power construal? To illustrate this issue, it is almost equally important to understand how people in powerful and powerless conditions construe power.

Our research aims to advance power theories in two significant ways. First, we extend the existing concept of power construal by proposing the power-nurtured power concept theory, which builds upon the work of Torelli and Shavitt (2010). While their theory highlights how culture shapes power construal, we argue that the immediate influence of power itself—specifically, the sense of powerful and powerless—is a more fundamental and direct factor in shaping how power is perceived and enacted. Our theory posits that powerful and powerless people construe power differently because of their position within the power hierarchy. This focus on power as a psychological force complements cultural explanations, offering a new lens through which power construal can be understood. Although culture undoubtedly plays a role, the power itself, independent of cultural context, is also and even a more critical factor in shaping people’s construal of power. While this phenomenon has been discussed philosophically (e.g., King, 2007; Machiavelli, 1532), our theory moves beyond cultural explanations to focus on the psychological impact of power and marks the first empirical demonstration of how power influences power construal through experimental investigation.

Second, our work transcends the prevailing one-sided focus on powerful people in the development of power theories (Schaerer et al., 2018) by considering the perspective of powerless people. Although some recent research (e.g., Ksenofontov and Becker, 2020; Pai et al., 2021; Schaerer et al., 2018; Schaerer et al., 2021) has investigated powerless people, most power-related research has concentrated on powerful people and treated powerless people as a mere comparison group. In fact, powerless people experience power everywhere. In a sense, they function as important monitors of how power is used, which can be reflected in their power construals. Thus, how powerless people construe power is indeed an essential part of power theory because it offers unique insights into how power operates from the subordinate side of power relationships. In this regard, our work may contribute to establishing a more holistic and nuanced theory of social power, surpassing the limitations inherent in one-sided perspectives (Schaerer et al., 2018).

Our focus lies in the sense of being powerful or powerless, underscoring that it is the sense rather than the factual existence of power that ultimately shapes people’s construals of power. For instance, even a manager with significant power may sense themselves as powerless when comparing upward (see Ten Brinke and Keltner, 2022). Furthermore, in this research, we operationalize power as the dichotomy between powerful and powerless people,^1^ stemming from our interest in examining how people construe power when in positions of power or a lack thereof, as opposed to analyzing a continuous spectrum of power hierarchy.

### 1.1 How does power influence power construal

Notably, once power is vested in people, it is no longer pure power but mixed with the characteristics and needs of power holders themselves (see Maner and Mead, 2010). While numerous studies have illuminated the negative or even antisocial consequences of power (e.g., Galinsky et al., 2006; Cislak et al., 2018; Weick, 2020), some research has contended that power can also induce a prosocial orientation in powerful people, prompting benevolent behavior toward others (Frieze et al., 2001; Gardner and Seeley, 2001; Greenberg, 1978; Lee and Tiedens, 2001). Chen et al. (2001) argued that powerful people’s prosocial orientations are functional, serving their strategic and self-interests. In fact, powerful people can sustain their power only by continuously meeting the needs of group members, necessitating the prioritization of others’ interests and relinquishing some self-centered power to maintain superior status or uphold prosocial values. At the very least, they need to be aware of and publicly express their concerns for others’ interests. Consequently, we posit that whether driven by genuine concern for others’ interests, functional considerations or a combination of the two, powerful people are cognizant of the importance of publicly expressing continuous concern for others.

Regarding powerless people, the inherent power imbalance (Boehm, 1999; Van Vugt et al., 2008) results in them constantly being influenced by the intentions of powerful people. Powerlessness may trigger anxiety, which in turn motivates powerless people to seek social control over others (Fiske et al., 1996). However, powerless people lack the tangible power to control others, but this attempt may increase their anxiety and lead them to think of power contradictorily. Some researchers have argued that occupying a high position in a power hierarchy can be both corruptive and ennobling (Cislak et al., 2018). On the one hand, powerful people recognize the necessity of continuously meeting the needs of group members; on the other hand, vast empirical literature in social psychology highlights the antisocial effects of power (Cislak et al., 2018). It is, therefore, unsurprising that powerless people experience power complexly. Overall, we hypothesize that, compared with people in powerless conditions, people in powerful conditions tend to construe power with a more socialized orientation than with a personalized orientation (H1).

### 1.2 Mechanism underlying the effect of power on power construal

We assume that psychological entitlement may be the mechanism underlying the effect of power on power construal. Psychological entitlement is a sense that one deserves more and is more important than others (Campbell et al., 2004; Fiske, 2010; Grubbs and Exline, 2016). This construct reflects a key psychological process through which power influences how people perceive and construe power. Entitlement arises when people feel that their contributions or roles warrant greater resources or recognitions (Fiske, 2010). Naumann et al. (2002) further argued that entitlement perceptions rest on the principle of reciprocity. Building on this, we posit that entitlement serves as a bridge linking power to power construal by shaping people’s interpretations of power dynamics, making it a theoretically grounded mediator in this research.

Regarding the relationship between power and psychological entitlement, previous research revealed that when people believe they have provided an essential good to another entity, they can exaggerate the significance of their contributions, making them feel entitled (Graffin et al., 2013; Harris and Schaubroeck, 1988). Webster et al. (2022) further reported that because those high in power are more capable of protecting and regulating their high performance, they often possess strong feelings of psychological entitlement (Webster et al., 2022). Piff (2014) argued that the sociocultural environments of social-class groups shape their levels of entitlement (Piff, 2014) and higher class may be associated with increased entitlement and narcissism (e.g., Kraus et al., 2012; Piff et al., 2010; Piff, 2014; Snibbe and Markus, 2005; Stephens et al., 2007). Although social class and power are distinct concepts, with social class reflecting a combination of factors such as work prestige, family wealth, and education (Kraus et al., 2012), they are closely related in how they influence people’s perceptions and behaviors. While we acknowledge that power and social class are not the same, past studies have frequently explored the overlap between the two, especially in understanding behaviors linked to entitlement. In this article, we focus on social power as a key determinant of psychological entitlement, distinct from the broader influence of social class, but recognize that the research on social class provides useful insights into understanding of how power shapes entitlement dynamics (e.g., De Cremer and Van Dijk, 2005; Kraus et al., 2012; Piff et al., 2010; Snibbe and Markus, 2005; Stouten et al., 2005).

The extant research further suggests that psychological entitlement can exist as a state-like phenomenon (Yam et al., 2017; Zitek et al., 2010), whereby a particular circumstance can propel people to adopt entitled states (e.g., Joplin et al., 2019), which in turn can influence their subsequent attitudes and behaviors (Webster et al., 2022). Recent theorizing has established entitlement as related to specific grandiosity (e.g., leadership, assertiveness, thrill seeking, Krizan and Herlache, 2018), and the sense of entitlement orients the individual toward maintaining an enhanced status. Moreover, some researchers have also found that entitlement is closely associated with narcissism (Brown et al., 2009; Campbell et al., 2004; Exline et al., 2004), which is a multifaceted construct characterized by an inflated view of the self, a self-aggrandizing and dominant orientation toward others, increased grandiosity, and heightened feelings of uniqueness and individualism (Emmons, 1984; Raskin and Terry, 1988; Twenge et al., 2008).

On the one hand, the entitlement that powerful people may often possess is associated with a host of undesirable and antisocial behaviors, including greater selfishness and rule-breaking in the workplace, as well as less empathy and respect for others (Campbell et al., 2004; Yam et al., 2017; Zitek et al., 2010). Powerless people may often observe or experience these features in powerful people from the subordinate side of the power relationship. Thus, it is reasonable to think that powerless people are more likely to consider powerful people entitled and therefore construe power as less socialized. Recent research has argued that in the West (individualistic culture), power is conceptualized as personalized and reflects the influence of entitlement (Torelli et al., 2020).

On the other hand, although powerful people are associated with greater psychological entitlement (De Cremer and Van Dijk, 2005; Stouten et al., 2005), they may not be conscious of this themselves. However, they may understand that the term entitlement is linked to various psychological problems, such as anxiety (Tritt et al., 2010; Watson and Biderman, 1993), anger (Grubbs et al., 2013), depression (Trumpeter et al., 2008), lower self-esteem, and poorer adjustment (Ackerman et al., 2011). In fact, an important aspect of psychological entitlement is that entitled individuals have a consistently positive view of themselves (Snow et al., 2001). To maintain positive self-views in the face of contradictory evidence, people often distort their perceptions of reality in such a way to maintain a desirable self-image (Martinko and Gardner, 1987). Thus, it is reasonable to think that powerful people are more likely to consider themselves not entitled and to construe power as socialized. Accordingly, we hypothesize that psychological entitlement mediates the relationship between power and power construal (H2).

## 2 Overview of the current research

This research includes two studies to test the hypotheses above. Study 1 examines H1, concentrating on how people in the powerful and powerless conditions construe power, which serves to clarify the influence that power has on power construal. Study 2 examines H2, exploring whether psychological entitlement mediates the relationship between power and power construal.

We report how we determined our sample size, all data exclusions (if any), all manipulations, and all measures in the study. The raw data of this research is available on the Open Science Framework, DOI 10.17605/OSF.IO/3AFJ4. Data were analyzed using SPSS, version 25. Study 2’s design and its analysis were preregistered at https://aspredicted.org/4GR_RQK and https://aspredicted.org/rx7t-8p4x.pdf.

## 3 Study 1

Study 1 was conducted to examine H1. In this study, power was primed by a recall task adapted from Galinsky et al. (2003),^2^ and power construal was measured by a revised scale based on Torelli and Shavitt (2010).^3^

### 3.1 Materials and methods

#### 3.1.1 Participants

According to the data of previous studies, we used G*Power 3.1 (Faul et al., 2007) to calculate the sample size, and 278 participants were required from Prolific in the USA to obtain adequate power (1 − β > 0.8) to detect a medium effect (*d* = 0.30). Finally, the data of 261 participants (125 men, 129 women, 7 with no sex information; age: *M* = 42.41, *SD* = 14.73) were included in the analyses, and 17 were excluded because of failure of the attention check. The participants were randomly assigned to one of two conditions (power: powerful vs. powerless). The dependent variable is power construal. Using sensitivity power analysis in G*Power, with 261 participants, the smallest effect size we could detect at 80% power (*α* = 0.05) would be *d* = 0.31 ( $\eta_{p}^{2}$ = 0.024).

#### 3.1.2 Procedure

Participants engaged in a recall task to prime their power conditions. They then answered four questions about how they construe power, completed the manipulation check and provided demographic information.

#### 3.1.3 Manipulations of power

Drawing from Galinsky et al. (2003), participants were asked to describe a situation in which they were/would be powerful or powerless, as shown in Table 1. Participants were asked to stay in this session for at least 1 min and to describe the powerful/powerless situation with no less than 50 words.

**Table 1:** Manipulations used in Study 1.

Powerful	Powerless
Please recall a particular moment in which you had some kind of social power. You were able to control certain resources, or were in a position to influence and evaluate others. At that moment, you were the holder of the power and you determined how to use it. Please reflect on such a time, describing the events that unfolded and your feelings during that period.	Please recall a particular moment in which you did not have any kind of social power. You were not able to control certain resources, and were not in a position to influence and evaluate others. At the moment, you were not the holder the power and could not determine how to use it. Please reflect on such a time, describing the events that unfolded and your feelings during that period.

#### 3.1.4 Measures of power construal

Four items were compiled based on Torelli and Shavitt (2010; see Table 2). Participants rated their agreement with these items from 1 (totally disagree) to 7 (totally agree). We calculated the average of two items as the scores of personalized and socialized orientations of power.

**Table 2:** Items of power construal used in Study 1.

	Item
Personalized	Powerful people are more likely to use the power to pursue their self-centered goals. Powerful people are more likely to use the power to achieve their personal accomplishments.
Socialized	Powerful people are more likely to use the power to pursue prosocial goals. Powerful people are more likely to use the power to benefit others.

#### 3.1.5 Manipulation Check

Participants rated the extent to which they perceived having power over others in the given situation on a scale from 1 (no power at all) to 7 (a lot of power).

### 3.2 Results

Participants in the powerful condition (*M* = 5.59, *SD* = 1.00) perceived a significantly higher sense of power than participants in the powerless condition (*M* = 1.98, *SD* = 1.16), *t* (259) = 27.09, *95% confidence interval* [−3.88, −3.35], *p* < 0.001, *d* = 3.33, indicating that the manipulation of power conditions was successful.

**Figure 1:**
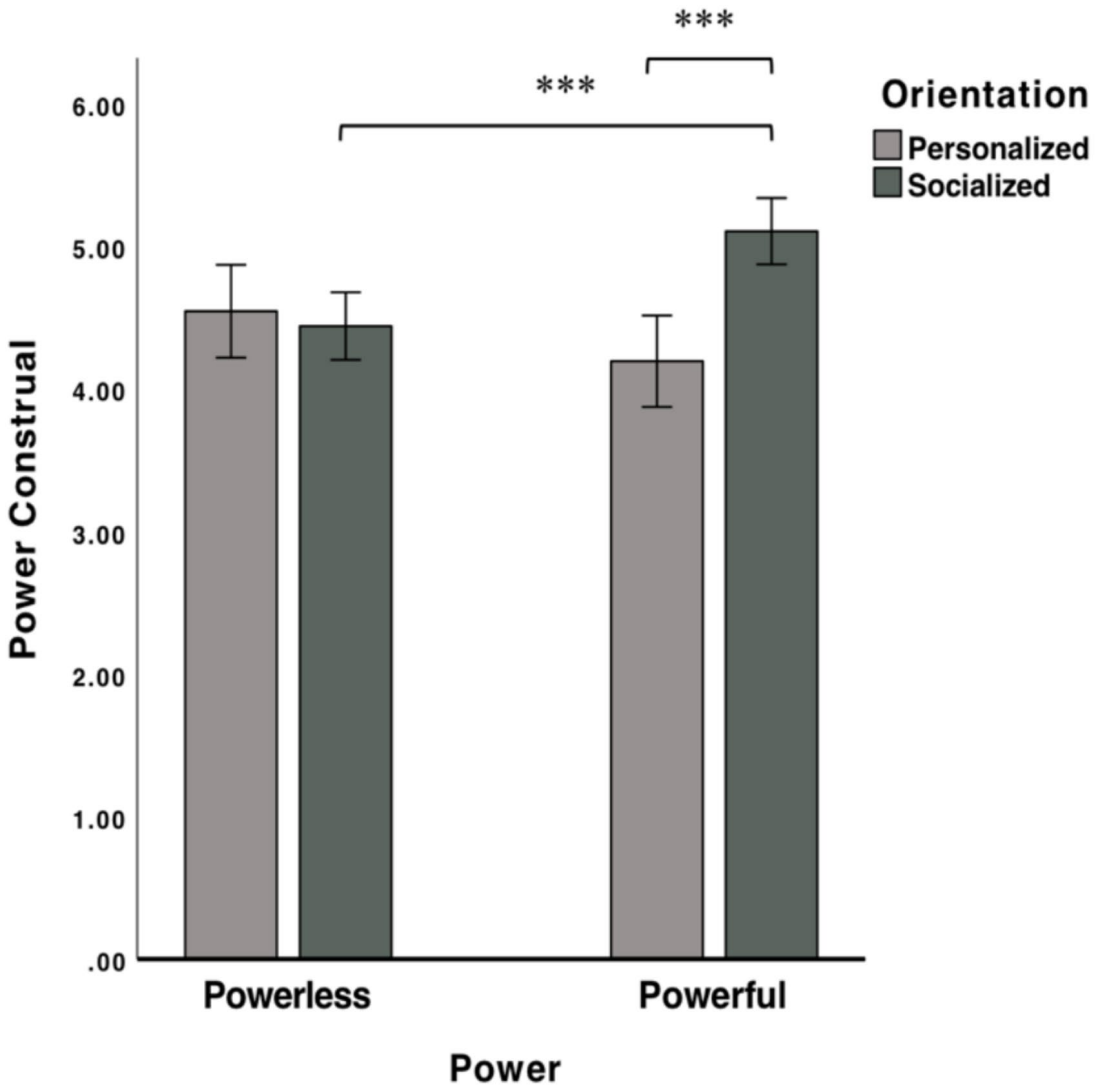
Study 1: power construal of people in powerful and powerless conditions (USA Sample). (1) error bars represent 95% confidence intervals; (2) ***p < 0.001.

A2 (power: powerful vs. powerless) × 2 (construal orientation: socialized vs. personalized) mixed ANOVA was conducted. As shown in Figure 1, a significant interaction effect of power and construal orientation emerged, *F* (1, 259) = 9.02, *p* = 0.003, $\eta_{p}^{2}$ = 0.034. Specifically, regardless of within group or between groups, participants in the powerful condition tended to construe power as socialized, and in the powerless condition, they tended to construe power as personalized. Within group, participants in the powerful condition construed power more in a socialized orientation (*M* = 5.08, *SD* = 1.25) than in a personalized orientation (*M* = 4.19, *SD* = 1.77), *F* (1, 259) = 15.96, *p* < 0.001, $\eta_{p}^{2}$ = 0.058, *Post hoc*: 1 − β = 0.98, whereas participants in the powerless condition construed power in a contradictory manner, with no significant difference between personalized (*M* = 4.52, *SD* = 1.90) and socialized (*M* = 4.46, *SD* = 1.43) orientations, *F* (1, 259) = 0.08, *p* = 0.784. Between groups, participants in the powerful condition construed power in a more socialized orientation (*M* = 5.08, *SD* = 1.25) than in the powerless condition (*M* = 4.46, *SD* = 1.43), *F* (1, 259) = 14.24, *p* < 0.001, $\eta_{p}^{2}$ = 0.052, *Post hoc*: 1 − β = 0.96, whereas participants in the powerful (*M* = 4.19, *SD* = 1.77) and powerless (*M* = 4.52, *SD* = 1.90) conditions construed power insignificantly differently in the personalized orientation, *F* (1, 259) = 2.11, *p* = 0.148. H1 is supported.

**Figure 2:**
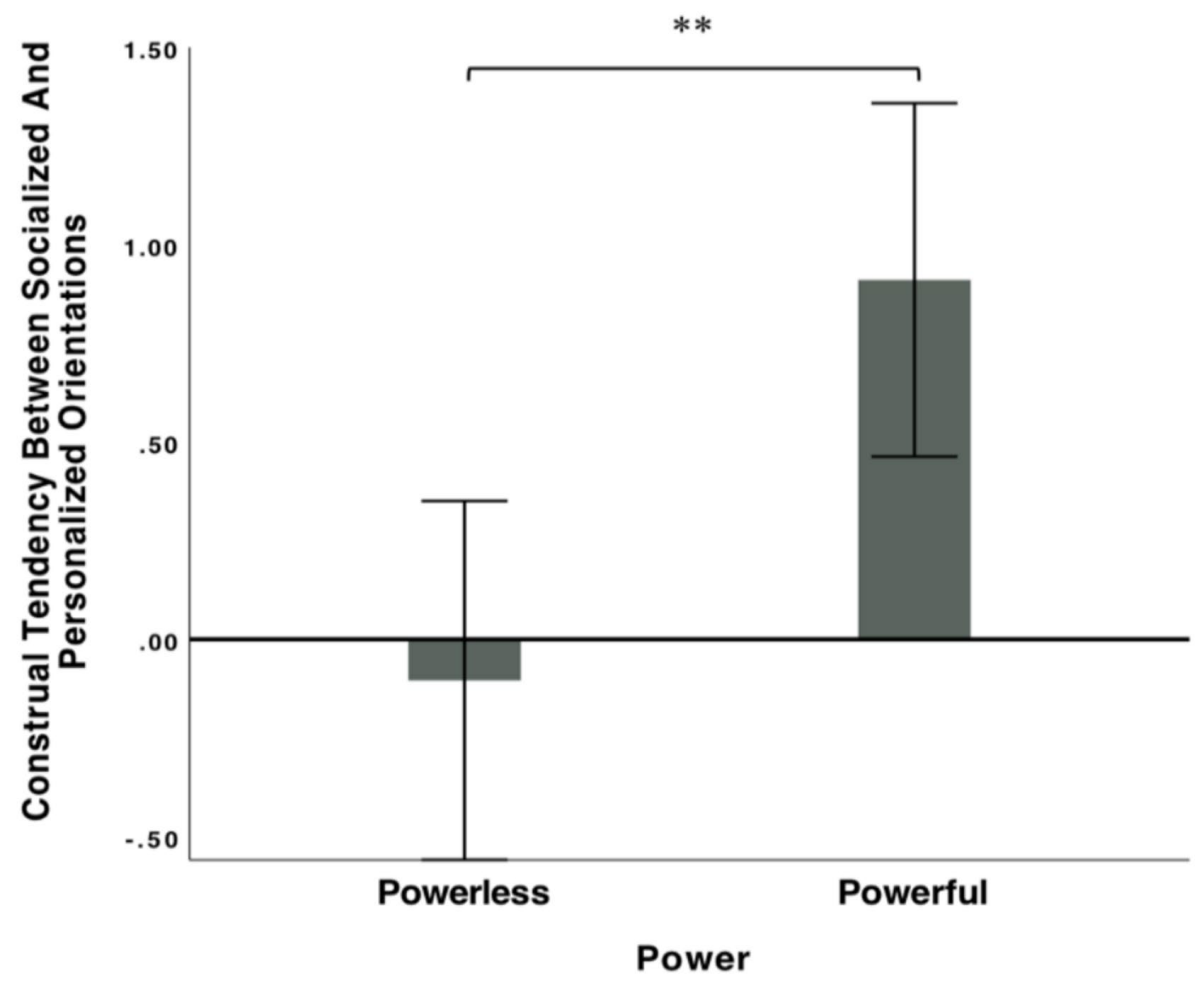
Study 1: construal tendency between personalized and socialized orientation of people in powerful and powerless conditions (USA Sample). (1) error bars represent 95% confidence intervals; (2) **p < 0.01.

To further explore participants’ construing tendencies between personalized and socialized orientations, we calculated the difference in scores for construing socialized and personalized orientations. Positive values indicate stronger inclinations toward construing power as socialized, whereas negative values indicate stronger inclinations toward construing power as personalized. An independent sample *t* test indicated that, as shown in Figure 2, participants in the powerful and powerless conditions construed power differently, *t* (259) = 3.00, *95% confidence interval* [−1.58, −0.33], *p* = 0.003, *d* = 0.37, *Post hoc*: 1 − β = 0.91. Specifically, compared with participants in the powerless condition (*M* = −0.06, *SD* = 2.73), participants in the powerful condition (*M* = 0.89, *SD* = 2.40) clearly construed power toward a socialized orientation rather than a personalized orientation.

Compared with participants in the powerless condition, participants in the powerful condition tended to construe power more as socialized rather than personalized, regardless of whether the original scores or the difference between socialized and personalized scores are used to calculate construal orientation.

## 4 Study 2

Study 2a and Study 2b aimed to further explore the mechanism underlying the effect of power on power construal. The hypothesis to be examined is that psychological entitlement mediates the relationship between power and power construal (H2).

### 4.1 Study 2a

#### 4.1.1 Materials and methods

##### 4.1.1.1 Participants

According to the data of previous studies, we used G*Power 3.1 (Faul et al., 2007) to calculate the sample size, and 278 participants were required to obtain adequate power (1 − β > 0.8) to detect a medium effect (*d* = 0.30). To be conservative, 360 participants were recruited from Prolific from the USA. The data of 3 participants were excluded from the analyses because of failure to complete the attention check. Finally, 357 participants (176 men, 178 women, 3 with no sex information; age: *M* = 38.94, *SD* = 12.07) were randomly assigned to one of two conditions (power: powerful vs. powerless). The dependent variable was power construal. Using sensitivity power analysis in G*Power, with 357 participants, the smallest effect size we could detect at 80% power (*α* = 0.05) would be *d* = 0.26. This study was preregistered at https://aspredicted.org/4GR_RQK.

##### 4.1.1.2 Procedure

Participants engaged in a recall task to prime their power conditions. They then completed the measure of psychological entitlement and answered four questions about how they construe power, completed the manipulation check and provided demographic information.

##### 4.1.1.3 Manipulations of power

Same as that in Study 1.

##### 4.1.1.4 Measure of entitlement

The measure of entitlement was adapted from the subscale of Self-Presentation Tactic Scale (Lee et al., 1999), as shown in Table 3. Participants rated the frequency of these items from 1 (very infrequently) to 7 (very frequently). We calculated the average of these items as the entitlement score. We also measured power maintenance and enhancement as potential competitive mediators, using another two subscales of Self-Presentation Tactic Scale (Lee et al., 1999). We take power maintenance and enhancement into account because power may increase holders’ motivation to maintain and increase the power gap between themselves and other group members, thereby protecting and entrenching their privileged position, whereas powerless people may be motivated to decrease the power gap (Maner and Mead, 2010). Thus, we suspect that power maintenance and enhancement may also function as mediators.

**Table 3:** Items of entitlement used in Study 2a.

	Item
Entitlement Cronbach’s *α* = 0.873	Powerful people claim credit for doing things they did not do. Powerful people point out the positive things they do which other people fail to notice. Powerful people tell people about their positive accomplishments. When working on a project with a group, powerful people make their contribution seems greater than it is. When telling someone about past events, powerful people claim more credit for doing positive things than was warranted by the actual events.

##### 4.1.1.5 Measures of power construal

Same as that in Study 1.

##### 4.1.1.6 Manipulation Check

Same as that in Study 1.

#### 4.1.2 Results

Participants in the powerful condition (*M* = 5.18, *SD* = 1.25) perceived a significantly greater sense of power than people in the powerless condition (*M* = 2.20, *SD* = 1.14), *t* (355) = 23.38, *95% confidence interval* [−3.23, −2.73], *p* < 0.001, *d* = 2.48, indicating that the manipulation of power conditions was successful.

**Figure 3:**
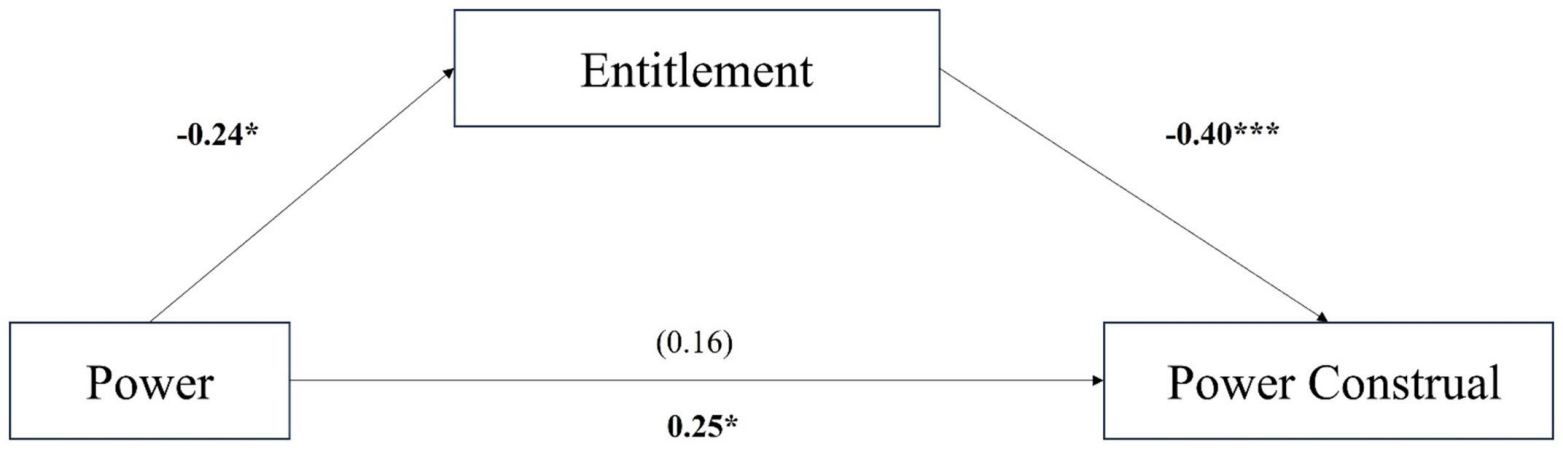
Study 2a: entitlement mediated the relationship between power and power construal (USA sample). (1) the statistics above the horizontal arrow indicate the effect of direct path (c’), and the statistics below indicate the effect of total path (c). (2) *p < 0.05, ***p < 0.001.

To facilitate the mediation model test, we calculated the difference between socialized and personalized scores as the dependent variable. Participants in the powerless condition (*M* = 5.60, *SD* = 0.98) were more likely to perceive that powerful people engage in entitlement behaviors than participants in the powerful condition (*M* = 5.34, *SD* = 1.18), *t* (355) = 2.34, *95% confidence interval* [0.04, 0.50], *p* = 0.020, *d* = 0.24. We then conducted mediation analyses (Model 4, based on 5,000 bootstrap samples) using the procedures (Process in SPSS) outlined by Preacher and Hayes (2008) to examine whether power had indirect associations with power construal through entitlement, as shown in Figure 3. Specifically, power had an indirect effect on power construal through entitlement (*Effect* = 0.10*, SE* = 0.04*, 95% confidence interval* [0.02, 0.18]). The mediating effects of power maintenance (*Effect* = 0.03, *SE* = 0.04, *95% confidence interval* [−0.02, 0.13]) and enhancement (*Effect* = 0.03, *SE* = 0.04, *95% confidence interval* [−0.05, 0.12]) were not significant. In summary, we found that psychological entitlement mediated the relationship between power and power construal, but power maintenance and enhancement did not mediate this relationship. H2 is supported.

### 4.2 Study 2b

#### 4.2.1 Materials and methods

##### 4.2.1.1 Participants

According to the calculation in Study 2a, 278 participants were required to obtain adequate power (1 − β > 0.8) to detect a medium effect (*d* = 0.30). Finally, 277 participants (130 men, 143 women, 4 with no sex information; age: *M* = 37.38, *SD* = 26.23) were randomly assigned to one of two conditions (power: powerful vs. powerless). The dependent variable was power construal. Using sensitivity power analysis in G*Power, with 277 participants, the smallest effect size we could detect at 80% power (*α* = 0.05) would be *d* = 0.30. This study was preregistered at https://aspredicted.org/rx7t-8p4x.pdf.

##### 4.2.1.2 Procedure

Same as that in Study 2a.

##### 4.2.1.3 Manipulations of power

Same as that in Study 1.

##### 4.2.1.4 Measure of entitlement

The measure of entitlement was adapted from Scale of State Psychological Entitlement (Webster et al., 2022), adapted from Campbell et al. (2004), as shown in Table 4. Participants rated the frequency of these items from 1 (very infrequently) to 7 (very frequently). We calculated the average of these items as the entitlement score.

**Table 4:** Items of entitlement used in Study 2b.

	Item
Entitlement Cronbach’s *α* = 0.946	Power holders feel they are just more deserving than others. Power holders feel great things should come to them. Power holders feel that if they were on the Titanic, they would deserve to be on the first lifeboat! Power holders demand the best because they feel they are worth it. Power holders feel they do not necessarily deserve special treatment. Power holders feel they deserve more things in their life. Power holders feel that people like them deserve an extra break now and then. Power holders feel that things should go their way. Power holders feel entitled to more of everything.

##### 4.2.1.5 Measures of power construal

Same as that in Study 1.

##### 4.2.1.6 Manipulation Check

Same as that in Study 1.

#### 4.2.2 Results

Participants in the powerful condition (*M* = 4.95, *SD* = 1.22) perceived a significantly greater sense of power than people in the powerless condition (*M* = 1.79, *SD* = 1.16), *t* (275) = −22.02, *95% confidence interval* [−3.44, −2.88], *p* < 0.001, *d* = −2.66, indicating that the manipulation of power conditions was successful.

**Figure 4:**
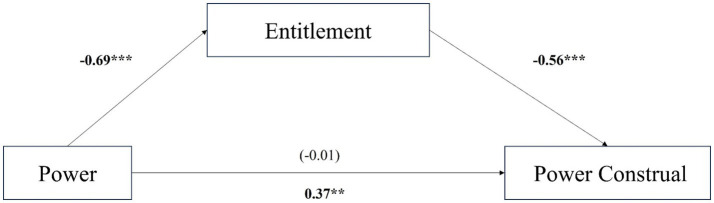
Study 2b: entitlement mediated the relationship between power and power construal (USA sample). (1) the statistics above the horizontal arrow indicate the effect of direct path (c’), and the statistics below indicate the effect of total path (c). (2) **p < 0.01, ***p < 0.001.

To facilitate the mediation model test, we calculated the difference between socialized and personalized scores as the dependent variable. Participants in the powerless condition (*M* = 4.82, *SD* = 1.27) were more likely to perceive that powerful people engage in entitlement behaviors than participants in the powerful condition (*M* = 5.65, *SD* = 0.98), *t* (275) = 6.13, *95% confidence interval* [0.57, 1.11], *p* < 0.001, *d* = 0.73. We then conducted mediation analyses (Model 4, based on 5,000 bootstrap samples) using the procedures (Process in SPSS) outlined by Preacher and Hayes (2008) to examine whether power had indirect associations with power construal through entitlement, as shown in Figure 4. Power had an indirect effect on power construal through entitlement (*Effect* = 0.39, *SE* = 0.07, *95% confidence interval* [0.26, 0.52]). In summary, we found that psychological entitlement mediated the relationship between power and power construal, but power maintenance and enhancement did not mediate this relationship. H2 is supported.

## 5 General discussion

This article examines how people in powerful and powerless conditions construe power. Three studies establish that people in powerful and powerless conditions exhibit distinct power construal and that psychological entitlement mediates the relationship between power and power construal. Specifically, people in powerful condition tend to construe power in a socialized orientation, whereas powerless people tend to construe power as a paradox, which is caused by their different senses of psychological entitlement. Our research reveals two interesting phenomena, termed the powerful people prosocial gloss and the powerless people paradox.

Powerful people prosocial gloss is the phenomenon in which people in powerful conditions tend to construe power in a socialized orientation, which is motivated by their psychological entitlement. The reasons for considering the phenomenon in this manner are as follows. First, despite this positive construal, empirical evidence from the field of social psychology consistently reports many negative and even antisocial effects of power (e.g., Anderson and Brion, 2014; Cislak et al., 2018; Galinsky et al., 2006; Weick, 2020; Zimbardo, 1973). Previous research suggests that the negative consequences of power arise because it affords power holders more freedom to act in alignment with their self-interests (e.g., Guinote, 2007; Smith and Trope, 2006; Whitson et al., 2013). This freedom allows them to pursue their own preferences and goals (Galinsky et al., 2008; Guinote, 2007; Smith and Trope, 2006), contributing to the observed negative effects of power. Briefly, on the one hand, based on our findings, powerful conditions lead people to construe power as socialized; on the other hand, based on previous findings, powerful conditions grant people freedom to act as their own interests. Powerful people’s construals of power and their behaviors are inconsistent. Thus, their socialized construals of power are more likely a prosocial gloss. Second, we examined competitive mediating models of entitlement, power maintenance and enhancement to explain the reasons underlying the effect of power on power construal. However, we found a mediating effect of psychological entitlement but not the other factors. That is, the motivation that powerful people possess to widen the power gap between themselves and powerless people (Winter, 1973; Chen et al., 2001; McClelland, 1987) does not always lead to their socialized construals of power; rather, their motivations to inflate their self-views, or narcissism (Brown et al., 2009; Campbell et al., 2004; Exline et al., 2004), that powerless people observe from powerful people lead to their socialized construals of power. Although socialized construal of power may not directly promote powerful people’s self-views, it directly promotes their image of power, through which powerful people’s self-views may increase.

In addition, we term this phenomenon powerful people prosocial gloss to emphasize that the socialized orientation is evident in construal rather than in action. In this sense, powerful people’s socialized construals of power can be linked to a form of deception. Powerful people tend to cheat (Lammers et al., 2010; Lammers et al., 2011). We refrain from using the term “cheating” and instead opt for “prosocial gloss” to emphasize that this positive portrayal remains in words (i.e., in construal not in action) and may be unconscious (e.g., powerful people may not perceive themselves entitled) and does not vary significantly from case to case, as cheating might. Moreover, as in-group members, people in powerful conditions may construe power in ways that are influenced by self-enhancement bias. We hope future research will further explore this potential association.

Our findings also reveal another intriguing phenomenon known as the powerless people paradox. This paradox by which people in powerless conditions view power as both personalized and socialized because they sense powerful people’s entitlement while the socialized construals from powerful people are simultaneously suffused in their lives. On the one hand, the vulnerability to exploitation of people in powerless conditions (Maner and Mead, 2010) contributes to their desires that powerful people will use power in a prosocial rather than self-serving manner. On the other hand, this optimism is shattered when powerless people realize that power holders frequently prioritize self-interest over prosocial concerns (see McClelland, 1987; Winter, 1973, 1993), and their socialized construals of power are just a gloss. Fundamentally speaking, the disjunction between socialized power desirability and personalized power perception gives rise to the powerless people paradox.

Our work advances culturally nurtured power concept theory (Torelli and Shavitt, 2010) to a more nuanced understanding termed “power-nurtured power concept theory.” We reveal that people’s sense of having (i.e., powerful) and lacking power (i.e., powerless) significantly influences their power construals. This disparity is inherently tied to power itself. Specifically, people in powerful conditions consistently exhibit a prosocial gloss, while people in powerless conditions grapple with a powerless paradox.

The findings of our work point toward several crucial avenues for future research and implications for understanding the dynamics of power construal. First, two of our studies engaged participants in independent responses without face-to-face interaction or collaborative work between powerful and powerless people. Recognizing the essential impact of situational context on human behavior (March, 1995; Messick, 1999), it is imperative for future research to delve into the influence of situational factors. Exploring varied interactive situations may elucidate nuanced differences in power construal.

Second, our findings predominantly focus on power construal in cognitive processes. Further research should extend its focus to investigating how people translate their power construals into behaviors. Despite abundant evidence highlighting the negative and even antisocial consequences of power (e.g., Anderson and Brion, 2014; Cislak et al., 2018; Galinsky et al., 2006; Weick, 2020; Zimbardo, 1973), our research offers a preliminary glimpse into the potential inconsistences between powerful people’s prosocial construals and their antisocial behaviors. To comprehensively understand the entire process—from power construal to power behavior—a more rigorous comparison within the same study and based on the same power group is warranted. This approach will facilitate a more nuanced exploration of the relationship between power construal and subsequent behavior, enhancing our understanding of the intricate dynamics at play between powerful and powerless people.

In conclusion, our research yields essential insights into the fundamental nature of power by identifying two important phenomena: the powerful people prosocial gloss and the powerless people paradox. While power may be overshadowed by the powerful people prosocial gloss, the powerless people paradox reveals the deceptive aspect of the prosocial gloss. Furthermore, our work may extend the culturally nurtured power concept theory to power-nurtured power concept theory, suggesting that people’s construals of power differ with their sense of power (i.e., have or lack power).

## Data Availability

The datasets presented in this study can be found in online repositories. The names of the repository/repositories and accession number(s) can be found in the article/supplementary material.

## Footnotes

^1^This dichotomous operationalization has several advantages. It allows for a clearer comparison between the sense of presence and absence of power, focusing the analysis on how people with or without power (rather than in different power hierarchies) construe power. The distinct categories of powerful and powerless enables a more straightforward interpretation of the effect of power *per se*, but a continuous power spectrum might be more complex than a clear effect of power. This approach is consistent with previous research, such as Guinote (2007), Galinsky et al. (2006), and Galinsky et al. (2008), which have commonly employed a dichotomous operationalization of power to investigate its psychological and behavioral effects. And, we also acknowledge the importance of a continuous power spectrum for a more nuanced understanding of power consequences and look forward to further exploration in future research.

^2^Considering that the original materials used by Galinsky et al. (2003) were designed for an undergraduate student sample, whereas our sample was collected online, with a broader range of educational backgrounds, we acknowledged greater variability in the educational level of participants. To ensure that participants could easily understand the concept of social power, we adapted the power priming materials to be more colloquial, making them more accessible and comprehensible to a wider range of participants with varying educational backgrounds.

^3^Given the lack of an existing measurement scale for power construal, we based our measurement items on the definitions of personalized and socialized power proposed by Torelli and Shavitt (2010). We extracted two items for each orientation, ensuring that these items accurately captured the core features associated with each orientation of power construal, thereby maintaining the validity and consistency of the measurement.
